# Antibacterial activities of the methanol extract, fractions and compounds from *Elaeophorbia drupifera* (Thonn.) Stapf. (Euphorbiaceae)

**DOI:** 10.1186/s12906-016-1509-y

**Published:** 2017-01-07

**Authors:** Igor K. Voukeng, Blaise K. Nganou, Louis P. Sandjo, Ilhami Celik, Veronique P. Beng, Pierre Tane, Victor Kuete

**Affiliations:** 1 Department of Biochemistry, Faculty of Science, University of Dschang, P.O. Box 67, Dschang, Cameroon; 2 Department of Chemistry, Faculty of Science, University of Dschang, Dschang, Cameroon; 3 Department of Pharmaceutical Sciences, CCS, Universidade Federal de Santa Catarina, Florianópolis, 88040-900 Santa Catarina Brazil; 4 Department of Chemistry, Faculty of Science, Anadolu University, Eskişehir, Turkey; 5 Department of Biochemistry, Faculty of Science, University of Yaounde I, Yaoundé, Cameroon

**Keywords:** Antibacterial, Crude extract, Compounds, *Elaeophorbia drupifera*, Fractions, Multi-drug resistance

## Abstract

**Background:**

*Elaeophorbia drupifera* (Thonn.) Stapf. (Euphorbiaceae) is used in Cameroonian folk medicine to treat several ailments including bacterial-related diseases such as skin infections. In this study, the methanol extract from the leaves (EDL), fractions (EDLa-d), sub-fractions EDLc1-7 and EDLc31-35 as well as isolated compounds were tested for their antimicrobial activities against a panel of Gram-negative and Gram-positive bacteria including multidrug resistant (MDR) phenotypes.

**Methods:**

The broth microdilution method was used to determine the minimal inhibitory concentration (MIC) and minimal bactericidal concentration (MBC) of the above samples; column chromatography was used for the fractionation and purification of the leaves extract whilst the chemical structures of compounds were determined using spectroscopic techniques.

**Results:**

Phytochemical investigation lead to the isolation of a mixture (1:3) of stigmasterol and *β*-sitosterol (**1** + **2**), euphol (**3**), sitosterol*-O-β-*_*D*_ *-*xylopyranoside (**4**), 3,3′,4′-tri-*O-*methylellagic acid (**5**), a mixture (1:1) of afzelin and quercetin-3-*O-β*-_D_-xylopyranoside (**6** + **7**), 3,3′,4′-tri-*O*-methylellagic acid 4-*O-β*-_D_-glucopyranoside (**8**), ellagic acid-4-*O-β*-xylopyranoside-3,3′,4′-trimethyl ether (**9**) from EDLc. Crude extract and fractions displayed selective activities with MIC values ranged from 32 to 1024 μg/mL for EDL against 84.9% of the 33 tested bacteria, 93.9% for EDLc, 69.7% for EDLb, 33.4% for EDLa and 0.03% for EDLd. MIC values ranged from 16 to 1024 μg/mL were obtained with EDLc3 and EDLc4 on all tested bacteria meanwhile other sub-fractions displayed selective activities. MIC value of 32 μg/mL was obtained with fractions EDLa against *Escherichia coli* AG100, EDLc against *Enterobacer aerogenes* ATCC13048 and EA298. For sub-fractions obtained from EDLc, the lowest MIC value of 16 μg/mL was recorded with EDLc3 against *Staphylococcus aureus* MRSA11. A corresponding value of 8 μg/mL against *Providencia stuartii* NAE16 was recorded with EDLc33 obtained from further fractionation of EDLc3. EDLc3 had MIC values below 100 μg/mL against all tested bacteria. Compound **5** as well as the mixture (1:1) of **6** and **7** inhibited the growth of all the tested bacteria with MICs ranged from 64 to 256 μg/mL.

**Conclusion:**

*Elaeophorbia drupifera* is a potential source of phytomedicine to tackle MDR bacteria. Sub-fraction EDLc3 was more active than all isolated compounds and deserves further investigations to develop natural drug to combat Gram-negative, Gram-positive bacteria and otherwise MDR phenotypes.

**Electronic supplementary material:**

The online version of this article (doi:10.1186/s12906-016-1509-y) contains supplementary material, which is available to authorized users.

## Background

Despite the progress in antibiotherapy, the fight against bacterial infections still constitute a major concern worldwide. The antibacterial fight is seriously challenged by the development of of multi-drug resistant (MDR) phenotypes [1]. Scientists should take into account the resistance issue when investigating natural products for their antimicrobial potential. In regards of the biodiversity of plant kingdom, evidenced-based botanicals appear to be undeniable source of medicine to fight bacterial resistance [2, 3]. Recently, several bioactive plants against MDR bacteria were reported. Some of these include *Erythrina sigmoidea* [4], *Cinnamomum zeylanicum, Aframomum citratum*, *Paullinia pinnata* [5, 6], *Allanblackia gabonensis*, *Combretum molle* [7] and *Harungana madagascariensis* [8]. In our continous search of antibacterials from plants used traditionnally to manage microbial infections, we targeted *Elaeophorbia drupifera* (Thonn.) Stapf. (Euphorbiaceae). The plant is used in traditional medicine to treat skin infections, Guinea worm [9] as well as hypertension and diabetes [10]. Leaves extract was reported to moderately inhibit HIV-1 and HIV-2 proviral DNA copying [11], and to have relaxant effect on vascular smooth muscle on rats [12]. The leaves methanol extract of this plant also showed good cytotoxic activity towards leukemia CCRF-CEM and MDA-MB231 breast cancer cell lines [13]. Previous phytochemical investigations of the plant led to the isolation of triterpenoids and steroids [14, 15]. In the present study, the bioguided fractionation was undertaken for depth investigation of the antibacterial activity of methanolic extract of *Elaeophorbia drupifera* leaves’.

## Methods

### General procedure

Mass spectral data [Electrospray ionization mass spectrometry (ESI-MS)] were measured on a Waters Synapt HDMS spectrometer. NMR Spectra were recorded with an Agillent spectrometer at 400 MHz. Chemical shifts (*δ*) were quoted in parts per million (ppm) from the internal standard tetramethylsilane (TMS). Deuterated dimethyl sulfoxide (DMSO-*d*_*6*_), was used as solvent for the NMR experiments. Column chromatography was performed on silica gel Merck 60 F_254_ [(0.2–0.5 mm) and (0.2–0.063 mm)] 70–230 and 230–400 mesh (Darmstadt, Germany). Pre-coated silica gel 60 F_254_ thin layer chromatography (TLC) plates (Merck, Germany) were used for monitoring fractions and spots were detected with UV light (254 and 365 nm) and then sprayed with 50% sulphuric acid (H_2_SO_4_) followed by heating to 100 °C.

### Plant material and extraction

The leaves of *Elaeophorbia drupifera* were collected in Dschang, West Region of Cameroon (5°27′N 10°04′E) in January 2014. The plant was identified at the National Herbarium (Yaoundé, Cameroon) where a voucher specimen was deposited under the reference number 57644/HNC [roots, leaves, bark]. The powder leaves of *E. drupifera* (1300 g) was soaked in methanol (MeoH; 5 L) for 48 h. After filtration and removal of the solvent using a rotary evaporator under reduced pressure, 233 g of crude extract (EDL) was obtained.

### Isolation and purification of bioactive compounds from the leaves extract of *E. drupifera*

Part of crude extract (213 g) was dissolved in methanol-water (9:1 v/v), followed by a liquid-liquid fractionation using *n*-hexane (Hex), dichloromethane (CH_2_Cl_2_), and ethyl acetate (EtOAc) respectively. This afforded four fractions: 65 g EDLa (Hex), 48 g EDLb (CH_2_Cl_2_), 91 g EDLc (EtOAc), and 12 g residual fraction (EDLd). Of these fractions, EDLc shows the best antibacterial activity and was therefore subjected to further purification processes.

Fraction EDLc (85 g) was subjected to column chromatography (CC) over silica gel and eluted with increasing gradient of *n*-Hex-EtoAc and EtOAc – MeoH solvent mixtures. 167 Fractions of 300 mL each were collected; based on TLC results, they were combined into seven new sub- fractions tagged EDLc1 (7.5 g); EDLc2 (3.4 g); EDLc3 (8.5 g); EDLc4 (18.6 g); EDLc5 (17.3 g); EDLc6 (16.1 g) and EDLc7 (12.9 g). EDLc1 and EDLc7 displayed poor antibacterial effects and were not further investigated. EDLc2 (3.2 g) was subjected to CC over silica gel eluting with mixture of *n*-Hex-CH_2_Cl_2_ mixtures of increasing polarity. 69 new sub-frs of 100 mL each were collected and combined into 4 other new sub-fractions; they were labelled EDLc21 (0.3 g); EDLc22 (1.2 g); EDLc23 (0.25 g) and EDLc24 (0.5 g). Sub-fractions 18–30 (EDLc22) was further purified by silica gel column chromatography eluting with n-hexane-Acetone (95:5) isocratic, to afford an amorphous solid identified and mixture (1:3) of stigmasterol and *β*-Sitosterol (**1 + 2;** 18 mg) and another white powder identified as euphol (**3;** 22 mg).

EDLc3 (7.5 g) was subjected to silica gel CC eluting with mixture solvents of *n*-Hex-EtOAc of increasing polarity. 141 sub- fractions of 100 mL of each were collected and pooled into 5 sub-fractions: EDLc31 (0.6 g); EDLc32 (1.3 g); EDLc33 (1.9 g); EDLc34 (1.4 g); EDLc35 (0.9 g). The sub-fraction EDLc33 was subjected to further sephadex LH20 purification with *n*-Hex-CH_2_Cl_2_-MeOH (9:2:0.5) mixture. Two new sub-fractions named EDL331 (0.4 g) and EDL332 (0.9 g) were obtained. EDL332 was further subjected to another silica gel CC eluting with dichloromethane- acetone (98:2) isocratic system to afford a white powder identified sitosterol 3-*O-β*-_D_-xylopyranoside (**4;** 405.2 mg) and a brownish powder identified 3,3′,4′-tri-*O*-methylellagic acid (**5;** 8 mg).

EDLc4 (18.5 g) was subjected to CC over silica gel eluting with *n*-Hex-acetone mixtures and MeOH of increasing polarity. 108 sub-fractions of 100 mL each were collected and regrouped based on their TLC profile into 4 sub-fractions named EDLc41 (3.8 g), EDLc42 (4.1 g), EDLc43 (3.6 g) and EDLc44 (2.8 g). Compound **5** (17 mg) was obtained in sub-fractions (EDLc41 – 44).

Sub-fraction EDLc5 (17.2 g) was subjected to silica gel CC eluting with a gradient of CH_2_Cl_2_-EtOAc and EtOAc-MeOH of increasing polarity, and was collected in 143 new sub-fractions of 100 mL each were collected. They were further pooled upon TLC analysis into five sub-fraction named EDLc51 (3.6 g), EDLc52 (2.2 g), EDLc53 (2.4 g), EDLc54 (1.9 g) and EDLc55 (3 g). EDLc53 and EDLc54 were further subjected to Sephadex LH20 CC eluting with *n*-Hex- CH_2_Cl_2_-MeOH (7:4:0.5) mixture to afford a yellowish gum identified a mixture (1:1) of 3-*O*-rhamnopyranosyl kaemferol and 3-*O*- xylopyranosyl quercetin (**6 + 7**; 18 mg).

Sub-fraction EDLc6 (16 g) was subjected to silica gel CC eluting with mixture of CH_2_Cl_2_-acetone and acetone-MeOH of increasing polarity. 119 new sub-fractions of 100 mL of were collected and combined based on their TLC profiles into 4 sub-fractions: EDLc61 (3.3 g), EDLc62 (2.4 g), EDLc63 (1.2 g) and EDLc64 (4.6 g). EDLc62 afforded a white powder identified as 3,3′,4′-tri-*O*-méthylelleargic acid 4-*O-β*-_D_-glucopyranoside (**8**; 15.3 mg) and another white powder, ellagic acid-4-*O-β*-xylopyranoside-3,3′,4′-trimethyl ether (**9**; 30 mg).

### Antimicrobial assays

#### Chemicals for antimicrobial assay

The reference antibiotic (RA) used against bacteria was chloramphenicol ≥ 98% (Sigma-Aldrich, St. Quentin Fallavier, France) meanwhile the bacterial growth indicator was *p*-iodonitrotetrazolium chloride ≥ 97% (INT, Sigma-Aldrich).

#### Microbial strains and culture media

A panel of 33 bacteria belonging to Gram-negative and Gram-positive bacteria were investigated in this work. They included sensitive and resistant strains of *Escherichia coli, Enterobacter aerogenes, Enterobacter cloacae, Klebsiella pneumoniae, Pseudomonas aeruginosa* and *Providencia stuartii* (Gram-negative bacteria) as well as *Staplylococcus aureus* (Gram-positive bacteria). They were obtained clinically and from the American Type Culture Collection (ATCC). Their bacterial features were previously reported (Additional file 1: Table S1) [6]. Mueller Hinton Agar (Sigma) was used to activate the microorganisms whilst Mueller Hinton broth (MHB; Sigma) was used for antibacterial assays [16].

#### INT colorimetric assay for MIC and MBC determinations

The determinations MIC and MBC on the tested bacteria were monitored by the rapid INT colorimetric assay according to described methods [17] with some modifications [18, 19]. The test samples and RA were dissolved in dimethylsulfoxide (DMSO)/MHB. The final concentration of DMSO was lower than 2.5% and does not affect the microbial growth. The solution obtained was then added to MHB, and serially diluted two fold (in a 96- wells microplate). The bacterial concentration was 1.5 × 10^6^ CFU/mL. The plates were incubated at 37 °C for 18 h. The assay was repeated thrice. Wells containing MHB, 100 μL of inoculum and DMSO to a final concentration of 2.5% served as negative control. The MIC of samples was detected after 18 h incubation at 37 °C, following addition (40 μL) of 0.2 mg/mL of INT and incubation at 37 °C for 30 min as the lowest sample concentration that prevented the color change of the medium and exhibited complete inhibition of microbial growth [17]. The MBC was determined by adding 50 μL aliquots of the preparations, which did not show any growth after incubation during MIC assays, to 150 μL of MHB. These preparations were incubated at 37 °C for 48 h. The MBC was regarded as the lowest concentration of samples, which did not produce a color change after addition of INT as mentioned above [20, 21].

## Results

### Structural determination

**Fig. 1:**
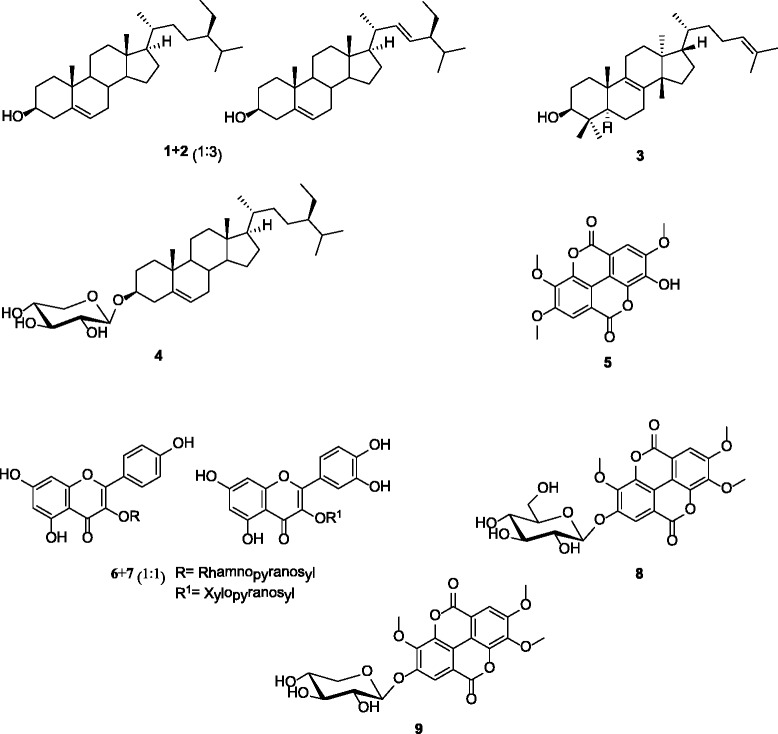
Chemical structures of compounds isolated from the leaves of *Elaoephorbia drupifera*. Mixture of stigmasterol and *β*-sitosterol (**1** + **2**); euphol (**3**); sitosterol*-O-β-*_*D*_ *-*xylopyranoside (**4**); 3,3′,4′-tri-*O-*methylellagic acid (**5**); mixture of afzelin and quercetin-3-*O-β*-_D_-xylopyranoside (**6** + **7**); 3,3′,4′-tri-*O*-methylellagic acid 4-*O-β*-_D_-glucopyranoside (**8**); ellagic acid-4-*O-β*-xylopyranoside-3,3′,4′-trimethyl ether (**9**)

The chemical structures of compounds from the leaves of *E. drupifera* were elucidated using physical and NMR data and comparison with literature. The isolated compounds were identified as the mixture (1:3) of stigmasterol and *β*-sitosterol (**1** + **2**) [22]; euphol C_30_H_50_O (**3**; m.p. 113.8–114.5 ^o^C; *m/z* 426; [α]_D_ +31 (*c* 0.2, CH_2_Cl_2_)) [23], sitosterol*-O-β-*_*D*_ *-*xylopyranoside C_34_H_58_O_5_ (**4**; m.p. 271–273 ^o^C; *m/z* 546; [α]_D_ – 50.1 (c 0.9, DMSO-*d*_*6*_)) [24], 3,3′,4′-tri-*O-*methylellagic acid C_17_H_12_O_8_ (**5**; m.p. 287–288.5 ^o^C; *m/z* 344) [25], the mixture (1:1) of afzelin and quercetin-3-*O-β*-_D_-xylopyranoside (**6** + **7**) [26, 27]; 3,3′,4′-tri-*O*-methylellagic acid 4-*O-β*-_D_-glucopyranoside C_23_H_22_O_13_ (**8**; m.p. 260-262 °C; *m/z* 506) [28] and ellagic acid-4-*O-β*-xylopyranoside-3,3′,4′-trimethyl ether C_22_H_20_O_12_ (**9**; m.p. 196.5–198.1 °C; *m/z* 476) [29] (Fig. 1). Extracts, fractions, compounds and the mixtures of two compounds identified in the leaves of *E. drupifera* were tested for their antimicrobial activities on a panel of Gram-negative and Gram-positive bacteria. The results are reported in Tables 1, 2, 3 and 4.

**Table 1:** MIC and MBC (in μg/mL) of crude extract, fractions of *E. drupifera* leaves and chloramphenicol against the panel of 33 bacteria

Bacterial strains	Tested samples and MICs MIC and MBC in parenthesis (in μg/mL)					
	Crude extract	Fractions				Reference drug
	EDL	EDLa	EDLb	EDLc	EDLd	CHL
*Escherichia coli*						
ATCC8739	-	64 (1024)	128 (1024)	64 (1024)	-	2 (128)
ATCC10536	256 (-)	-	-	128 (1024)	-	<2 (64)
AG100	1024 (-)	32 (512)	-	128 (512)	-	8 (128)
AG100A	512 (-)	1024 (-)	1024 (-)	256 (1024)	512 (-)	˂2 (128)
AG100A_TET_	1024 (-)	1024 (-)	1024 (-)	-	-	32 (-)
AG102	512 (-)	256 (-)	256 (1024)	256 (-)	-	64 (-)
MC4100	256 (-)	-	-	512 (-)	-	16 (-)
W3110	512 (-)	256 (1024)	128 (1024)	128 (-)	-	2 (-)
*Pseudomonas aeruginosa*						
PA01	-	1024 (-)	1024 (-)	256 (-)	-	32 (-)
PA124	-	1024 (-)	-	128 (-)	-	128 (-)
*Enterobacter aerogenes*						
ATCC13048	-	-	-	32 (512)	-	4 (32)
EA-CM64	512 (-)	-	512 (1024)	1024 (-)	-	256 (-)
EA3	1024 (-)	-	512 (-)	64 (512)	-	256 (-)
EA27	512 (-)	-	-	512 (-)	-	32 (-)
EA289	512 (-)	1024 (-)	512 (-)	128 (1024)	-	64 (-)
EA298	512 (-)	-	256 (1024)	32 (128)	-	128
*Providencia stuartii*						
NEA16	512 (-)	-	256 (512)	256 (-)	-	32 (256)
ATCC29916	1024 (-)	-	256 (1024)	256 (512)	-	16 (256)
PS2636	512 (-)	512 (-)	256 (-)	64 (512)	-	16 (256)
PS299645	1024 (-)	-	-	64 (128)	-	64 (-)
*Klebsiella pneumoniae*						
ATCC11296	512 (-)	-	1024 (-)	128 (512)	-	8 (256)
KP55	512 (-)	1024 (-)	256 (1024)	128 (512)	-	32 (256)
KP63	512 (-)	1024 (-)	1024 (-)	128 (512)	-	32 (-)
K24	512 (-)	-	-	-	-	64 (256)
K2	1024 (-)	-	512 (1024)	64 (512)	-	8 (256)
*Enterobacter cloacae*						
BM47	-	-	256 (-)	1024 (-)	-	256 (-)
BM67	512 (-)	-	512 (-)	256 (1024)	-	-
BM94	512 (-)	-	1024 (-)	1024 (-)	-	128 (-)
*Staphylococcus aureus*						
ATCC 25923	128 (1024)	-	1024 (-)	512 (-)	-	8 (128)
MRSA 3	1024 (-)	-	-	1024 (-)	-	16 (-)
MRSA 4	512 (-)	-	-	1024 (-)	-	64 (-)
MRSA 11	512 (-)	-	1024 (-)	512 (-)	-	32 (-)
MRSA 12	512 (-)	-	256 (1024)	128 (1024)	-	16 (256)

Tested samples were crude methanol extract of *E. drupifera* (EDL), hexane fraction (EDLa), dichloromethane fraction (EDLb), ethyl acetate fraction (EDLc), residual fraction (EDLc), and chloramphenicol (CHL); -: MIC or MBC values above 1024 μg/mL

**Table 2:** MIC and MBC (in μg/mL) of sub-fractions of ethyl acetate fraction EDLc against the panel of 26 bacteria

Bacterial strains	Sub-fractions, MIC and MBC in parenthesis (in μg/mL)						
	EDLc1	EDLc2	EDLc3	EDLc4	EDLc5	EDLc6	EDLc7
*Escherichia coli*							
ATCC8739	1024 (-)	512 (-)	64 (-)	512 (-)	512 (-)	-	-
ATCC 10536	-	1024 (-)	32 (-)	64 (-)	512 (-)	1024 (-)	1024 (-)
AG100A	-	512 (-)	64 (256)	256 (-)	-	-	-
AG102	-	128 (-)	32 (256)	256 (-)	64 (512)	1024 (-)	-
W3110	-	-	16 (-)	64 (-)	512 (-)	1024	-
*Pseudomonas aeruginosa*							
PA 01	-	128 (-)	64 (-)	512 (-)	1024 (-)	1024 (-)	1024 (-)
PA 124	-	1024 (-)	64 (-)	256 (-)	512 (-)	1024 (-)	1024 (-)
*Enterobacter aerogenes*							
ATCC13048	512 (-)	256 (-)	128 (256)	512 (-)	1024 (-)	-	1024 (-)
EA-CM64	-	1024 (-)	64 (256)	512 (-)	-	-	-
EA289	-	128 (-)	32 (-)	64 (-)	512 (-)	1024 (-)	512 (-)
EA27	1024 (-)	1024 (-)	128 (-)	256 (-)	512 (-)	-	1024 (-)
*Providencia stuartii*							
NEA16	1024 (-)	512 (-)	64 (-)	256 (-)	-	-	-
ATCC29916	-	256 (-)	64 (-)	256 (-)	512 (-)	-	-
PS2636	512 (-)	128 (-)	16 (-)	64 (-)	256 (-)	1024 (-)	512 (-)
PS299645	128 (-)	128 (-)	16 (-)	256 (-)	512 (-)	1024 (-)	512 (-)
*Klebsiella pneumoniae*							
ATCC11296	1024 (-)	512 (-)	64 (-)	256 (-)	512 (-)	-	-
KP55	-	128 (-)	32 (-)	512 (-)	1024 (-)	512 (-)	1024 (-)
KP63	-	1024 (-)	64 (-)	512 (-)	512 (-)	-	1024 (-)
K2	256 (-)	64 (-)	16 (-)	64 (-)	-	-	512 (-)
*Enterobacter cloacae*							
BM47	-	256 (1024)	64 (256)	256 (-)	512(-)	-	-
BM67	-	512(-)	64 (256)	256 (-)	1024 (-)	-	-
BM94	-	1024(-)	32 (256)	64 (-)	1024 (-)	-	1024 (-)
*Staphylococcus aureus*							
ATCC25923	-	-	64 (512)	512 (-)	1024 (-)	-	-
MRSA 3	-	1024 (-)	64 (256)	256 (-)	512 (-)	-	-
MRSA 4	-	256 (-)	32 (256)	256 (-)	512 (-)	-	-
MRSA 11	-	1024 (-)	16 (-)	128 (-)	256 (-)	1024 (-)	1024 (-)

-: MIC or MBC values above 1024 μg/mL

**Table 3:** MIC and MBC (in μg/mL) activities of sub-fractions EDLc3 against the panel of 24 bacteria

Bacterial strains	Sub-fractions, MIC and MBC in parenthesis (in μg/mL)				
	EDLc31	EDLc32	EDLc33	EDLc34	EDLc35
*Escherichia coli*					
ATCC8739	- (-)	256 (-)	64 (-)	256 (-)	512 (-)
ATCC 10536	256 (-)	64 (-)	64 (-)	32 (-)	128 (-)
AG100A	128 (-)	64 (-)	32 (-)	128 (512)	256 (-)
AG102	128 (-)	128 (-)	32 (1024)	256 (1024)	1024 (1024)
*Pseudomonas aeruginosa*					
PA 01	256 (-)	32 (-)	256 (1024)	32 (1024)	64 (-)
PA 124	-	-	-	-	-
*Enterobacter aerogenes*					
ATCC13048	-	256 (512)	512 (-)	128 (-)	256 (512)
EA-CM64	-	512 (-)	256 (-)	256 (1024)	256 (-)
EA289	128 (-)	32 (-)	64 (-)	32 (-)	64 (-)
EA27	-	128 (-)	256 (-)	512 (-)	256 (-)
*Providencia stuartii*					
NEA16	-	32 (-)	8 (-)	32 (-)	128 (1024)
ATCC29916	512 (-)	64 (-)	512 (-)	64 (-)	128 (-)
PS2636	128 (-)	32 (-)	128 (-)	16 (-)	64 (-)
PS299645	-	128 (-)	512 (-)	1024 (-)	1024 (-)
*Klebsiella pneumoniae*					
ATCC11296	512 (-)	256 (-)	-	64 (-)	128 (-)
KP63	-	32 (512)	64 (1024)	128 (1024)	256 (-)
K2	16 (-)	128 (-)	64 (-)	16 (-)	32 (-)
*Enterobacter cloacae*					
BM47	-	256 (-)	1024 (-)	128 (-)	256 (-)
BM67	-	256 (512)	64 (-)	64 (-)	128 (1024)
BM94	-	64 (-)	256 (-)	1024 (-)	1024 (-)
*Staphylococcus aureus*					
ATCC25923	-	256 (-)	256 (-)	128 (-)	256 (-)
MRSA 3	-	256 (-)	64	64 (1024)	128 (1024)
MRSA 4	-	256 (-)	256	64 (-)	128 (-)
MRSA 11	256 (-)	64 (-)	128	16 (1024)	64 (1024)

-: MIC or MBC values above 1024 μg/mL

**Table 4:** MIC (in μg/ml) of compounds isolated from of *E. drupifera* leaves against the panel of 14 bacteria

Bacterial strains	Compounds and MIC (in μg/ml)			
	3	5	6 + 7	8
*Escherichia coli*				
ATCC8739	-	128	64	256
ATCC10536	-	128	128	256
AG102	-	128	64	64
*Pseudomonas aeruginosa*				
PA 01	-	128	128	-
PA 124	256	128	128	64
*Enterobacter aerogenes*				
ATCC13048	-	128	64	128
EA-CM64	-	128	128	128
*Providencia stuartii*				
ATCC29916	-	128	128	256
PS2636	-	64	64	256
*Klebsiella pneumoniae*				
ATCC11296	-	128	128	256
KP55	-	128	128	128
*Staphylococcus aureus*				
ATCC 25923	-	128	64	64
MRSA3	-	256	128	64
MRSA4	-	256	128	256

Tested compounds were a mixture (1:3) of stigmasterol and *β*-sitosterol (**1** + **2**); euphol (**3**); sitosterol*-O-β-*_*D*_ *-*xylopyranoside (**4**); 3,3′,4′-tri-*O-*methylellagic acid (**5**); mixture 1:1 of afzelin and quercetin-3-*O-β*-_D_-xylopyranoside (**6** + **7**); 3,3′,4′-tri-*O*-methylellagic acid 4-*O-β*-_D_-glucopyranoside (**8**); ellagic acid-4-*O-β*-xylopyranoside-3,3′,4′-trimethyl ether (**9**). No MBC was detected at 256 μg/mL for all compounds; MIC values were >256 μg/mL for **1 + 2, 4** and **9**

### Antibacterial activity

Crude leaves extract (EDL), fractions EDLa-d and chloramphenicol were tested on a panel of 33 bacteria. The results summarized in Table 1 reveal selective activities with MIC values ranged from 32 to 1024 μg/mL for EDL against 28/33 (84.9%) tested bacteria as well as EDLc, EDLb, EDLa and EDLd respectively against 31/33 (93.9%), 23/33 (69.7%), 12/33 (33.4%) and 1/33 (0.03%) tested bacteria. MIC values below 1024 μg/mL were also recorded with chloramphenicol on 32/33 (97.0%) tested bacteria. MBC values below1024 μg/mL were obtained with EDLa-d as well as their mother extract EDL and RA on some of the tested bacterial strains. However, the recorded values were generally high. Table 2 reports the MIC and MBC values of sub-fractions from EDLc (EDLc1–7) against a panel of 26 bacteria. It appears that MIC values ranged from 16 to 1024 μg/mL were obtained with EDLc3 and EDLc4 on all tested bacteria meanwhile other sub-fractions displayed selective activities. Their inhibitory effects were noted on 24/26 (92.3%), 22/26 (84.6%), 13/26 (50.0%), 10/26 (38.5%) and 8/26 (30.8%) tested bacteria. Further investigations of EDLc3 yielded five new sub-fractions (EDL31 to EDL35) with selective activities (Table 3). MIC values ranged from 8 to 1024 μg/mL on 23/24 (95.8%) tested bacteria for EDLc32, EDLc34 and EDLc35. It should be noted that MBC values noted with EDL31-EDL35 were generally above 1024 μg/mL. A keen look of data from Tables 1 and 3 indicated that the ratio MBC/MIC were generally above 4. The antibacterial activities of the isolated compounds and the mixtures of two compounds are compiled in Table 4. Compound **5** as well as the mixture (1:1) of **6** and **7** inhibited the growth of all the 14 tested bacteria with MIC values ranged from 64 to 256 μg/mL. Compounds **3** and **8** inhibited respectively the growth of 1/14 (7.1%) and 13/14 (92.9%) tested bacteria meanwhile **4, 9** as well as the mixture (1:3) of **1** and **2** were not active at up to 256 μg/mL.

## Discussion

In the present study, we identified 9 compounds amongst which were 4 terpenoids (**1–4**), 2 flavonoid glycosides (**6** and **7**), 3 ellagic acid derivatives (**5, 8** and **9**). Among terpenoids were steroids (**1** and **2**), a steroid glucoside (**4**) and a triterpenoid (**3**). The isolation of compounds such as euphol (**3**), tirucallol, euphorbol, ingenol elaeophorbate, epitaraxerol, taraxerone, friedelin, lup-20(29)-en-3-one or lupenone, lupeol, olean-12-ene-3-one, olean-12-ene-3-ol, elaeophorbate in *E. drupifera* was previously reported [14, 15]. However, in the present work, fewer compounds as well as other not previously isolated ones were isolated, probably due to the fact that the purification was guided by the antibacterial activity and hence all fractions and sub-fractions were not explored. According to established criteria, the antibacterial activity of a plant extract is considered to be significant when MIC is below 100 μg/mL, moderate when 100 μg/mL < MIC < 625 μg/mL or low when MIC > 100 μg/mL [30, 31]. Therefore, the antibacterial activity of the leaves extract (EDL) of *E. drupifera* could be considered as moderate, as MIC values below 625 μg/mL were obtained on the majority of the tested bacteria (Table 1). However, fractionation of EDL afforded more active samples, and the antibacterial effects of EDLc, EDLc3, EDLc4, EDLc32 to EDLc35 could be considered important. In fact, the lowest MIC value of 32 μg/mL was obtained with fractions EDLa against *Escherichia coli* AG100, EDLc against *Enterobacer aerogenes* ATCC13048and EA298. For sub-fractions obtained from EDLc, the lowest MIC value of 16 μg/mL was noted with EDLc3 against *Staphylococcus aureus* MRSA11. A corresponding value of 8 μg/mL against *Providencia stuartii* NAE16 was recorded with EDLc33 obtained from further fractionation of EDLc3. These data highlight the increase of activity with consecutive fractionation of extracts and also demonstrate the good antibacterial potential of *E. drupifera.* Imporantly, the MIC values obtained with the best sub-fractions EDLc3 against *Pseudomonas aeruginosa* PA124, *E. aerogenes* EA289, *Providencia stuartii* PS29964, *Enterobacter cloacae* BM47, BM67, BM94, *S. aureus* MRSA4 (Table 2) and EDLc33 against *P. stuartii* NAE16 (Table 2) were lower than those of chormaphenicol. It should also be highlighted that EDLc3 had MIC values below 100 μg/mL against all the 26 tested bacteria (Table 2). The ratio MBC/MIC obtained were generally above 4, indicating that the studied extracts as well as the active fractions mostly exerted bacteriostatic effects [32–34]. Also, MIC and MBC values of the reference drug chloramphenicol were also very high (>64 μg/mL) on several pathogens, confirming that most of the bacterial strains used were MDR phenotypes. The activity of compounds is significant when MIC < 10 μg/mL, moderate when 10 < MIC < 100 μg/mL and low when MIC > 100 μg/mL [30, 31]. On this basis, none of the compound or mixtures displayed significant antibacterial activity. Also the lowest MIC value of 64 μg/mL obtained with compound **5** was much more higher than the corresponding values for the most active fraction EDLc3 where it were isolated. This suggests that constituents of this fraction may exert synergistic effects. This also indicates that combating the tested bacteria with fractions and mostly EDLc3 could be more efficient than with isolated compounds. When regarding the structure-activity relationship, it appears that terpenoids were poorly or not active against the tested bacteria. This result is not suprising, as terpenoids are known to generally have poor antibacterial activity [30]. Amongst the three ellagic acid derivatives, compound **5** and the glucoside **8** had antibacterial activities contrary to **9**. Hence, it may be deduced that the substitution of a glucosyl- (**8**) group by the xylopyranosyl- group (**9**) cancels the antibacterial effect of the ellagic acid derivative **9**.

To the best of our knowledge, the identification of the antibacterial constituents of *E. drupifera* is being reported for the first time. However, the antibacterial activity of the leave of extract of the plant was reported on *Staphylococcus aureus* and *Streptococcus pyogenes* [9]*.* The present study therefore provides more information on the antibacterial potential of *E. drupifera* and identified the bioactive components of plant. Also, compound **5** was previously reported against a panel of sensitive Gram-positive and Gram-negative bacteria, with MIC values ranged from 9.76 to 156.25 μg/mL [18]. Data obtained in the present study (MIC ranged from 64 to 256 μg/mL) are in consistence with previous and also confirm the activity of compound **5** against MDR bacteria.

## Conclusions

The results of the present investigation are very interesting, taking in account the medical importance of the studied microorganisms. The most active fraction of the plant, identified as EDLc3 displayed significant antibacterial activity on a panel of 26 tested bacteria including both sensitive and MDR Gram-negative and Gram-positive bacteria. This fraction could therefore be useful in the management of bacterial infection including MDR phenotypes. The bioactive constituents of the plant include 3,3′,4′-tri-*O*-methylelleargic acid, 3-*O*-rhamnopyranosyl kaempherol, 3-*O*-rhamnopyranosyl quercetin and 3,3′,4′-tri-*O*-méthylelleargic acid 4-*O-β*-_D_-glucopyranoside.

## Additional file

Additional file 1:
**Table S1.** Bacterial strains used and their features. (DOCX 2625 kb)

## Abbreviations

1: Stigmasterol
2: *β*-sitosterol
3: Euphol
4: Sitosterol*-O-β-*_*D*_ *-*xylopyranoside
5: 3,3′,4′-tri-*O-*methylellagic acid
6: Afzelin
7: Quercetin-3-*O-β*-_D_-xylopyranoside
8: 3,3′,4′-tri-*O*-methylellagic acid 4-*O-β*-_D_-glucopyranoside
9: Ellagic acid-4-*O-β*-xylopyranoside-3,3′,4′-trimethyl ether
ATCC: American Type Culture Collection
CFU: Colony forming unit
DMSO: Dimethyl sulfoxide
*E. aerogenes*: *Enterobacter aerogenes*
*E. cloacae*: *Enterobacter cloacae*
*E. coli*: *Escherichia coli*
EDL: Methanol extract from the leaves of *Elaeophorbia drupifera*
EDLa-d: Fractionsfrom EDL
EDLc1-7: Sub-fractions from EDLc
H_2_SO_4_: Sulphuric acid
HNC: National Herbarium of Cameroon
INT: *p*-iodonitrotetrazolium chloride ≥ 97% (INT, Sigma-Aldrich).
*K. pneumoniae*: *Klebsiella pneumoniae*
MBC: Minimal bactericidal concentration
MDR: Multidrug resistant
MDR: Multi-drug resistant
MHB: Mueller Hinton Broth
MIC: Minimal inhibitory concentration
*P. aeruginosa*: *Pseudomonas aeruginosa*
*P. stuartii*: *Providencia stuartii*
RA: Reference antibiotic
TLC: Thin layer chromatography
