# Supplementary material for: Antibacterial activities of the methanol extract, fractions and compounds from Elaeophorbia drupifera (Thonn.) Stapf. (Euphorbiaceae)
Source: BMC Complement Altern Med. 2017 Jan 7;17:28. doi: 10.1186/s12906-016-1509-y (PMC5219710; doi:10.1186/s12906-016-1509-y)
Supplement: Additional file 1: — Table S1. Bacterial strains used and their features. (DOCX 2625 kb) [file 12906_2016_1509_MOESM1_ESM.docx]

**Antibacterial Activities of the Methanol Extract, Fractions and Compounds from *Elaeophorbia drupifera* (Thonn.) Stapf. (Euphorbiaceae)**

Igor K. Voukeng^1^, Blaise K. Nganou^2^, Louis P. Sandjo^3^, Ilhami Celik^4^, Veronique P. Beng^5^, Pierre Tane^2^ and Victor Kuete^1*^

*^1^ Department of Biochemistry, Faculty of Science, University of Dschang, Cameroon;*

*^2^ Department of Chemistry, Faculty of Science, University of Dschang, Cameroon;*

*3* *Department of Pharmaceutical Sciences, CCS, Universidade Federal de Santa Catarina, Florianópolis 88040-900, Santa Catarina, Brazil;*

^4^*Department of Chemistry, Faculty of Science, Anadolu University, Eskişehir, Turkey*

*^5^Department of Biochemistry, Faculty of Science, University of Yaounde I, Cameroon;*

**Corresponding authors:**

**Tel : (237) 77 35 59 27 ; Fax: (237) 22 22 60 18. P.O. Box 67 Dschang, Cameroon; E-mail:* [*kuetevictor@yahoo.fr*](mailto:kuetevictor@yahoo.fr) *(Prof. Dr. Victor Kuete)*

*Mixture of stigmasterol and β-sitosterol* (**1**+**2**).White amorphous solid, ^1^H NMR (400 MHz, CDCl_3_) δ 5.35 (H-6, *d*, *J* = 4.9 Hz), 5.12 (H-22, *m*), 5.01 (H-23, *m*), 3.52 (H-3, *m*), 2.27 (H-4, *m*), 1.01 (H-18, *s*), 0.81 (H-19, *s*); ^13^C NMR(100 MHz*,* CDCl_3_): *β*-*sitosterol*140.7(C-5), 121.7(C-6), 71.8 (C-3), 56.7(C-14), 55.9 (C-17), 50.1 (C-9),45.8(C-24), 42.3 (C-4), 42.3(C-13), 39.7 (C-12). *Stigmasterol*140.7 (C-5),121.7 (C-6), 71.8 (C-3), 56.8 (C-14), 56.0(C-17), 51.2 (C-24), 50.1(C-9), 42.3(C-4), 42.3(C-13), 39.8 (C-12) (De-Eknamkul and Potduang, 2003).

^1^H NMR (400 MHz, CDCl_3_)of the mixture **1** and **2**

^13^C NMR (100 MHz, CDCl_3_) of the mixture **1** and **2**

*Euphol* (**3**) white powder; m.p. 113.8-114.5 ^o^C; [α]_D_+31 (*c* 0.2, CH_2_Cl_2_);^13^C NMR (100 MHz, CDCl_3_)δ134.0 (C-8), 133.5 (C-9), 130.9 (C-25), 125.2 (C-24), 79.0 (C-3), 50.9 (C-5), 50.0 (C-14), 49.6 (C-17), 44.1 (C-13), 38.9 (C-4), 37.2 (C-10), 35.9 (C-20), 35.4 (C-22), 35.2 (C-1), 30.8 (C-12), 29.7 (C-15), 28.2 (C-16), 28.0 (C-28), 27.9 (C-2), 27.6 (C-7), 25.7 (C-27), 24.7 (C-23), 24.5 (C-30), 21.5 (C-11), 20.1 (C-19), 18.9 (C-6, C-21), 17.7 (C-26), 15.6 (C-18), 15.5 (C-29) (Gewali et al., 1990).

^1^H NMR (400 MHz, CDCl_3_)of compound **3**

^13^C NMR (100 MHz, CDCl_3_) of compound **3**

*Sitosterol-O-β-_D_-xylopyranoside* (**4**) white powder; m.p. 271-273 ^o^C; [α]_D_ – 50.1 (c 0.9, DMSO-*d_6_*);^13^C NMR (100 MHz, DMSO-*d_6_*) δ 140.9 (C-5), 121.6 (C-6), 77.3 (C-3), 56.6 (C-14), 55.9 (C-17), 50.0 (C-9), 50.0 (C-24), 45.6 (C-13), 42.3 (C-4), 37.3 (C-10), 36.6 (C-1), 35.9 (C-12), 33.8 (C-7), 33.4 (C-20), 31.8 (C-2), 31.8 (C-22), 29.7 (C-23), 29.1 (C-8), 28.2 (C-16), 25.8 (C-15), 24.3 (C-25), 23.0 (C-11), 21.4 (C-28), 20.2 (C-19), 19.5 (C-26), 19.4 (C-27), 19.0 (C-21), 12.2 (C-18), 12.1 (C-29),101.2 (C-1’),73.9 (C-2’)77.2(C-3’), 70.5 (C-4’), 61.5 (C-5’) (Alam et al., 2010).

^1^H NMR (400 MHz, DMSO-*d_6_*)of compound **4**

^13^C NMR (100 MHz, DMSO-*d_6_*) of compound **4**

*3,3’,4’-tri-O-methylellagic acid* (**5**) Brownish powder; m.p. 287-288.5 ^o^C; ^13^C NMR (100 MHz, DMSO-*d_6_*) δ159.0 (C-7), 158.8 (C-7’), 154.3 (C-4’), 153.1 (C-4), 142.0 (C-2), 141.3 (C-2’), 141.3 (C-3), 140.6 (C-3’), 113.0 (C-6), 112.4 (C-6’), 112.1 (C-1’), 111.7 (C-1), 110.0 (C-5), 107.9 (C-5’), 61.8 (MeO-3), 61.5 (MeO-3’), 57.2 (MeO-4’) (Abd El Azim et al., 2014).

^1^H NMR (400 MHz, DMSO-*d_6_*) of compound **5**

^13^C NMR (100 MHz, DMSO-*d_6_*) of compound **5**

Mixture of afzelin and quercetin-3-*O-β*-_D_-xylopyranoside (**6**+**7**) Yellow gum; ^13^C NMR (100 MHz, DMSO-*d_6_*):Afzelin δ 178.2 (C-4), 164.6 (C-7), 161.7 (C-5), 160.4 (C-4’), 157.7 (C-9), 156.9 (C-2), 134.6 (C-3), 120.9 (C-1’), 131.0 (C-2’, C-6’), 115.8 (C-3’, C-5’), 104.6 (C-10), 99.1 (C-6),94.1 (C-8),102.2 (C-1”), 71.0 (C-2”), 71.5 (C-3”), 72.9 (C-4”), 70.5 (C-5”), 17.8 (C-6”).Quercetin-3-O-β-D-xylopyranoside δ 178.1 (C-4),164.7 (C-7), 161.7 (C-5), 157.7 (C-9), 156.9 (C-2), 134.6 (C-3), 99.1 (C-6), 94.0 (C-8), 104.6 (C-10), 121.5 (C-1’), 110.0 (C-2’), 145.6 (C-3’), 148.9 (C-4’), 110.0 (C-2’), 121.1 (C-6’), 102.2 (C-1”), 71.6 (C-2”), 71.1 (C-3”), 70.7 (C-4”), 63.5 (C-5”) (Lee et al., 2014; Zhu et al., 2013).

^1^H NMR (400 MHz, DMSO-*d_6_*) of the mixture (**6**+**7)**

Magnified ^1^H NMR (400 MHz, DMSO-*d_6_*) of the mixture (**6**+**7**)

^13^C NMR (100 MHz, DMSO-*d_6_*) of the mixture (**6**+**7**)

3,3’,4’-tri-*O*-methylellagic acid 4-*O-β*-_D_-glucopyranoside (**8**) White powder; m.p. 260-262^o^C;^1^H (400 MHz, DMSO-*d_6_*) δ112.0 (C-1), 141.7 (C-2), 140.8 (C-3), 151.8 (C-4), 112.3 (C-5), 112.6 (C-6), 158.9 (C-7), 112.8 (C-1’), 141.7 (C-2’), 140.8 (C-3’), 154.2 (C-4’), 107.6 (C-5’), 114.1 (C-6’), 158.6 (C-7’), 61.2 (MeO-3), 61.5 (MeO-3’), 56.3 (MeO-4’), 101.7 (C-1”), 73.2 (C-2”), 77.7 (C-3”), 69.9 (C-4”), 78.1 (C-5”), 62.1 (C-6”) (Ye et al., 2007).

^1^H NMR (400 MHz, DMSO-*d_6_*) of compound **8**,


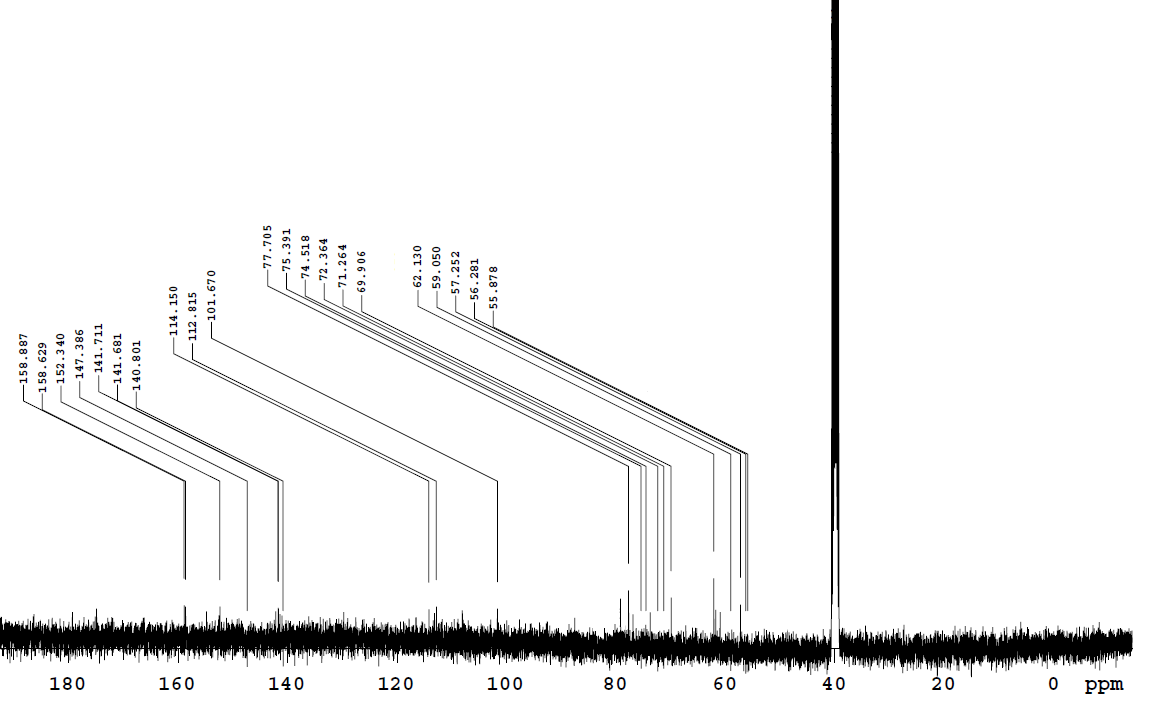


^13^C NMR (100 MHz, DMSO-*d_6_*) of compound **8**

Ellagic acid-4-*O-β*-xylopyranoside-3,3’,4’-trimethyl ether (**9**) White powder, m.p. 196.5-198.1^o^C;^13^C NMR (100 MHz, DMSO-*d_6_*)δ112.9 (C-1), 141.4 (C-2), 142.4 (C-3), 152.2 (C-4), 112.9 (C-5), 113.2 (C-6), 158.9 (C-7), 112.9 (C-1’), 141.4 (C-2’), 141.7 (C-3’), 154.8 (C-4’), 108.0 (C-5’), 114.3 (C-6’), 158.6 (C-7’), 61.7 (MeO-3), 62.2 (MeO-3’), 57.4 (MeO-4’),101.0 (C-1”), 75.1 (d, C-3”), 72.4 (C-2”), 68.6 (C-4”), 65.8 (C-5”) (Sinha et al., 1999).


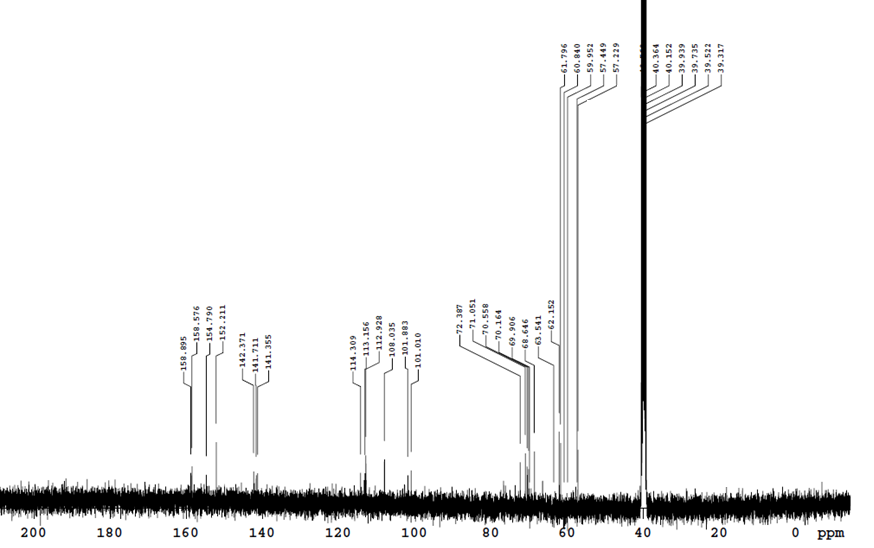


^13^C NMR (100 MHz, DMSO-*d_6_*) of compound **9**

**Table S1.** Bacterial strains used and their features

| Strains | Features and References |  |
| --- | --- | --- |
| *Escherichia coli* |  |  |
| ATCC10536 | Reference strain |  |
| AG100 | Wild-type *E. coli* K-12 | (Viveiros et al., 2005) |
| AG100A | AG100 *ΔacrAB*::KAN^R^ | (Kuete et al., 2010; Okusu et al., 1996; Viveiros et al., 2005) |
| AG100A_TET_ | Δ*acrAB* mutant AG100, with over-expressing  *acrF* gene ; TET^R^ | (Viveiros et al., 2005) |
| AG102 | Δ*acrAB* mutant AG100, owing *acrF* gene markedly over-expressed; TET^R^ | (Elkins and Mullis, 2007; Kuete et al., 2011) |
| MC4100 | Wild type *E. coli* | (Baglioni et al., 2003) |
| W3110 | Wild type *E. coli* | (Baglioni et al., 2003; Sar et al., 2005) |
| *Enterobacter aerogenes* |  |  |
| ATCC13048 | Reference strains |  |
| CM64 | CHL^R^ resistant variant obtained from ATCC13048 over-expressing the AcrAB pump | (Ghisalberti et al., 2005) |
| EA3 | Clinical MDR isolate; CHL^R^, NOR^R^, OFX^R^, SPX^R^, MOX^R^, CFT^R^, ATM^R^, FEP^R^ | (Mallea et al., 1998; Mallea et al., 2003) |
| EA27 | Clinical MDR isolate exhibiting energy-dependent norfloxacin and chloramphenicol efflux with KAN^R^ AMP^R^ NAL^R^ STR^R^ TET^R^ | (Mallea et al., 1998; Mallea et al., 2003) |
| EA289 | KAN sensitive derivative of EA27 | (Pradel and Pages, 2002) |
| EA294 | EA289 a*crA::*KAN^R^ | (Pradel and Pages, 2002) |
| EA298 | EA 289 *tolC::*KAN^R^ | (Pradel and Pages, 2002) |
| *Enterobacter cloacae* |  |  |
| BM94 | Clinical MDR isolates, CHL^R^ | (Fankam et al., 2011) |
| BM67 | Clinical MDR isolates, CHL^R^ | (Fankam et al., 2011) |
| BM47 | Clinical MDR isolates, CHL^R^ | (Fankam et al., 2011) |
| *Klebsiella pneumoniae* |  |  |
| ATCC12296 | Reference strains |  |
| KP55 | Clinical MDR isolate, TET^R^ , AMP^R^, ATM^R^, CEF^R^ | (Chevalier et al., 2000) |
| KP63 | Clinical MDR isolate, TET^R,^ CHL^R^, AMP^R,^ ATM^R^ | (Chevalier et al., 2000) |
| K24 | AcrAB-TolC, Laboratory collection of UNR-MD1, University of Marseille, France | (Fankam et al., 2011) |
| K2 | AcrAB-TolC, Laboratory collection of UNR-MD1, University of Marseille, France | (Fankam et al., 2011) |
| *Providencia stuartii* |  | (Tran et al., 2010) |
| NEA16 | Clinical MDR isolate, AcrAB-TolC |  |
| ATCC29916 | Clinical MDR isolate, AcrAB-TolC |  |
| PS2636 | Clinical MDR isolate, AcrAB-TolC |  |
| PS299645 | Clinical MDR isolate, AcrAB-TolC |  |
| *Pseudemonas aeruginosa* |  |  |
| PA 01 | Reference strains |  |
| PA 124 | MDR clinical isolate | (Lorenzi et al., 2009) |
| *Staphylococcus aureus* |  |  |
| ATCC25923 | Reference strain | (Paudel et al., 2012) |
| MRSA 3 | OFX^R^, KAN^R^, TET^R^, ERM^R^ resistant, clinical isolate |  |
| MRSA 4 | Clinical MDR isolate OFX^R^, KAN^R^, CHL^R^, CIP^R^ |  |
| MRSA 11 | Clinical MDR isolateOFX^R^, KAN^R^, ERM^R^, CIP^R^, IM/CS^R^ |  |
| MRSA 12 | Clinical MDR isolateOFX^R^, FLX^R^, KAN^R^, ERM^R^, IM/CS^R^ |  |

^a^AMP^R^, ATM^R^, CEF^R^, CFT^R^, CHL^R^,CIP^R^, ERM^R^, FEP^R^, FLX^R^, IM/CS^R^, KAN^R^, MOX^R^, OFX^R^, STR^R^, TET^R^, Resistance to ampicillin, aztreonam, cephalothin, cefadroxil, chloramphenicol, Ciprofloxacin, Erythromycin, cefepime,Flomoxef, Imipenem/ Cilastatin sodium, kanamycin, moxalactam, streptomycin, and tetracycline; MDR : Multidrug resistant.

**References**

Abd El Azim, M., El-Gerby M, A., Awad, A., 2014. Anti-Tumor, antioxidant and antimicrobial and the phenolic constituents of clove flower buds *(Syzygium aromaticum*) J Microbial BiochemTechnol 007, S8.

Alam, S., Ali, M., Alam, P., Shuaib, M., 2010. Phytochemical investigation of the seeds of *Butea monosperma Chemistry of Natural Compounds* 46, 44-48.

Baglioni, P., Bini, L., Liberatori, S., Pallini, V., Marri, L., 2003. Proteome analysis of *Escherichia coli* W3110 expressing an heterologous sigma factor. Proteomics 3, 1060-1065.

Chevalier, J., Pages, J.M., Eyraud, A., Mallea, M., 2000. Membrane permeability modifications are involved in antibiotic resistance in *Klebsiella pneumoniae*. Biochem Biophys Res Commun 274, 496-499.

De-Eknamkul, W., Potduang, B., 2003. Biosynthesis of beta-sitosterol and stigmasterol in *Croton sublyratus* proceeds via a mixed origin of isoprene units. Phytochemistry 62, 389-398.

Elkins, C.A., Mullis, L.B., 2007. Substrate competition studies using whole-cell accumulation assays with the major tripartite multidrug efflux pumps of *Escherichia coli*. Antimicrob Agents Chemother 51, 923-929.

Fankam, A.G., Kuete, V., Voukeng, I.K., Kuiate, J.R., Pages, J.M., 2011. Antibacterial activities of selected Cameroonian spices and their synergistic effects with antibiotics against multidrug-resistant phenotypes. BMC Complement Altern Med 11, 104.

Gewali, M., Hattori, M., Tezuka, Y., Kikuchi, T., Namba, T., 1990. Constituents of the latex of *Euphorbia antiquorum*. Phytochemistry 29, 1625-1628.

Ghisalberti, D., Masi, M., Pages, J.M., Chevalier, J., 2005. Chloramphenicol and expression of multidrug efflux pump in *Enterobacter aerogenes*. Biochem Biophys Res Commun 328, 1113-1118.

Kuete, V., Alibert-Franco, S., Eyong, K.O., Ngameni, B., Folefoc, G.N., Nguemeving, J.R., Tangmouo, J.G., Fotso, G.W., Komguem, J., Ouahouo, B.M., Bolla, J.M., Chevalier, J., Ngadjui, B.T., Nkengfack, A.E., Pages, J.M., 2011. Antibacterial activity of some natural products against bacteria expressing a multidrug-resistant phenotype. Int J Antimicrob Agents 37, 156-161.

Kuete, V., Ngameni, B., Tangmouo, J.G., Bolla, J.M., Alibert-Franco, S., Ngadjui, B.T., Pages, J.M., 2010. Efflux pumps are involved in the defense of Gram-negative bacteria against the natural products isobavachalcone and diospyrone. Antimicrob Agents Chemother 54, 1749-1752.

Lee, S.Y., So, Y.J., Shin, M.S., Cho, J.Y., Lee, J., 2014. Antibacterial effects of afzelin isolated from *Cornus macrophylla* on *Pseudomonas aeruginosa*, a leading cause of illness in immunocompromised individuals. Molecules 19, 3173-3180.

Lorenzi, V., Muselli, A., Bernardini, A.F., Berti, L., Pages, J.M., Amaral, L., Bolla, J.M., 2009. Geraniol restores antibiotic activities against multidrug-resistant isolates from gram-negative species. Antimicrob Agents Chemother 53, 2209-2211.

Mallea, M., Chevalier, J., Bornet, C., Eyraud, A., Davin-Regli, A., Bollet, C., Pages, J.M., 1998. Porin alteration and active efflux: two in vivo drug resistance strategies used by *Enterobacter aerogenes*. Microbiology 144 ( Pt 11), 3003-3009.

Mallea, M., Mahamoud, A., Chevalier, J., Alibert-Franco, S., Brouant, P., Barbe, J., Pages, J.M., 2003. Alkylaminoquinolines inhibit the bacterial antibiotic efflux pump in multidrug-resistant clinical isolates. Biochem J 376, 801-805.

Okusu, H., Ma, D., Nikaido, H., 1996. AcrAB efflux pump plays a major role in the antibiotic resistance phenotype of Escherichia coli multiple-antibiotic-resistance (Mar) mutants. J Bacteriol 178, 306-308.

Paudel, A., Hamamoto, H., Kobayashi, Y., Yokoshima, S., Fukuyama, T., Sekimizu, K., 2012. Identification of novel deoxyribofuranosyl indole antimicrobial agents. J Antibiot (Tokyo) 65, 53-57.

Pradel, E., Pages, J.M., 2002. The AcrAB-TolC efflux pump contributes to multidrug resistance in the nosocomial pathogen *Enterobacter aerogenes*. Antimicrob Agents Chemother 46, 2640-2643.

Sar, C., Mwenya, B., Santoso, B., Takaura, K., Morikawa, R., Isogai, N., Asakura, Y., Toride, Y., Takahashi, J., 2005. Effect of *Escherichia coli* wild type or its derivative with high nitrite reductase activity on in vitro ruminal methanogenesis and nitrate/nitrite reduction. J Anim Sci 83, 644-652.

Sinha, A., Taylor, W.H., Khan, I.H., McDaniel, S.T., Esko, J.D., 1999. Glycoside primers of *Psittacanthus cucullaris*. J Nat Prod 62, 1036-1038.

Tran, Q.T., Mahendran, K.R., Hajjar, E., Ceccarelli, M., Davin-Regli, A., Winterhalter, M., Weingart, H., Pages, J.M., 2010. Implication of porins in beta-lactam resistance of *Providencia stuartii.* J Biol Chem 285, 32273-32281.

Viveiros, M., Jesus, A., Brito, M., Leandro, C., Martins, M., Ordway, D., Molnar, A.M., Molnar, J., Amaral, L., 2005. Inducement and reversal of tetracycline resistance in Escherichia coli K-12 and expression of proton gradient-dependent multidrug efflux pump genes. Antimicrob Agents Chemother 49, 3578-3582.

Ye, G., Peng, H., Fan, M., Huang, C., 2007. Ellagic acid derivatives from the stem bark of *Dipentodon sinicus*. Chemistry of Natural Compounds 43, 125-127.

Zhu, Y., Liu, Y., Zhan, Y., Liu, L., Xu, Y., Xu, T., Liu, T., 2013. Preparative isolation and purification of five flavonoid glycosides and one benzophenone galloyl glycoside from Psidium guajava by high-speed counter-current chromatography (HSCCC). Molecules 18, 15648-15661.
